# Three-Dimensional Computer Model of the Right Atrium Including the Sinoatrial and Atrioventricular Nodes Predicts Classical Nodal Behaviours

**DOI:** 10.1371/journal.pone.0112547

**Published:** 2014-11-07

**Authors:** Jue Li, Shin Inada, Jurgen E. Schneider, Henggui Zhang, Halina Dobrzynski, Mark R. Boyett

**Affiliations:** Institute of Cardiovascular Sciences, University of Manchester, Core Technology Facility, Manchester, United Kingdom; Gent University, Belgium

## Abstract

The aim of the study was to develop a three-dimensional (3D) anatomically-detailed model of the rabbit right atrium containing the sinoatrial and atrioventricular nodes to study the electrophysiology of the nodes. A model was generated based on 3D images of a rabbit heart (atria and part of ventricles), obtained using high-resolution magnetic resonance imaging. Segmentation was carried out semi-manually. A 3D right atrium array model (∼3.16 million elements), including eighteen objects, was constructed. For description of cellular electrophysiology, the Rogers-modified FitzHugh-Nagumo model was further modified to allow control of the major characteristics of the action potential with relatively low computational resource requirements. Model parameters were chosen to simulate the action potentials in the sinoatrial node, atrial muscle, inferior nodal extension and penetrating bundle. The block zone was simulated as passive tissue. The sinoatrial node, crista terminalis, main branch and roof bundle were considered as anisotropic. We have simulated normal and abnormal electrophysiology of the two nodes. In accordance with experimental findings: (i) during sinus rhythm, conduction occurs down the interatrial septum and into the atrioventricular node via the fast pathway (conduction down the crista terminalis and into the atrioventricular node via the slow pathway is slower); (ii) during atrial fibrillation, the sinoatrial node is protected from overdrive by its long refractory period; and (iii) during atrial fibrillation, the atrioventricular node reduces the frequency of action potentials reaching the ventricles. The model is able to simulate ventricular echo beats. In summary, a 3D anatomical model of the right atrium containing the cardiac conduction system is able to simulate a wide range of classical nodal behaviours.

## Introduction

Accurate simulation of the generation and propagation of cardiac electrical activity requires detailed anatomical and electrophysiological models. A variety of heart anatomical models (human and animal) have been generated by various investigators. David et al. [1] generated a boundary-conforming mesh of the human atria comprised entirely of hexahedral elements. Bernus et al. [2] developed a human ventricular model, which reproduces geometry and fibre orientation in the right and left ventricles of the human heart. Several whole human heart models have been generated [3]–[5]. Aslanidi et al. [6] integrated a three-dimensional (3D) model of the human sinoatrial node (SAN) [7] into a 3D model of the whole atria dissected from the Visible Human dataset [8]. Apart from human heart models, a number of animal heart models have been generated. Nielsen et al. [9] developed a mathematical representation of the ventricular geometry and fibre orientation for the dog. Vetter and McCulloch [10] developed a 3D finite element model of rabbit ventricular geometry with fibre orientation. However, none of these models includes both the sinoatrial and atrioventricular (AVN) nodes. We have previously generated 3D anatomically-detailed models of the isolated SAN and isolated AVN of the rabbit [11], [12]. More recently, we have generated a 3D anatomically-detailed model of the rabbit heart, including the conduction system, using micro-CT [13], although at present the model is unsuitable for electrophysiological simulation. In this study, a 3D anatomically-detailed model of the right atrium of the rabbit heart, including the SAN and AVN and suitable for electrophysiological simulations, was generated based on magnetic resonance (MR) imaging.

There are two groups of electrophysiological models for simulation of the cardiac action potential. One comprises biophysically-detailed (ionic) models, and another comprises mathematical caricature (simplified) models. Based on patch clamp etc., a large number of biophysically-detailed models of the action potential in single cells from different regions of the heart have been developed [14]. For example, such models have been developed for: human atrial [15], [16] and ventricular [17]–[22] muscle; dog atrial [23], and ventricular [25]–[28] muscle; sheep Purkinje fibres [29], [30]; rabbit SAN [31]–[38], AVN [39], Purkinje fibres [40] and atrial [41]–[43] and ventricular [40], [44], [45] muscle; guinea-pig ventricular muscle [46]–[51]; and mouse SAN [52] and ventricular muscle [53]. Caricature models of the action potential include the cellular automaton model, coupled map lattices [54], lattices of coupled ordinary differential equations [55], FitzHugh-Nagumo models [56] and the Fenton-Karma model [57]. Action potential propagation through the heart can be simulated using caricature models as well as biophysically-detailed models. To investigate complex electrophysiological behaviour in the complex anatomical structure of the heart, the use of a set of caricature models is computationally more effective compared to the use of a set of biophysically-detailed models, as they enable testable predictions about heart electrophysiological behaviour to be made with relatively low computational resource.

The purpose of this study was to create a platform with an anatomically-detailed model of rabbit right atrium and a set of caricature action potential models to investigate nodal electrophysiology in health and disease. A 3D anatomically-detailed model of the rabbit right atrium with multiple objects, including the cardiac conduction system (SAN and AVN), was generated. This model can be used for education and research. The propagation of the action potential through the right atrium during sinus rhythm, atrial fibrillation and AVN reentry was simulated using this anatomically-detailed right atrium model, together with a set of Rogers-modified FitzHugh-Nagumo models as well as a cellular automaton model.

## Methods

### Ethics Statement

New Zealand White rabbits (1.5–2.5 kg) were sacrificed humanely according to the United Kingdom Animals (Scientific Procedures) Act 1986; in addition, the investigation conformed with the Guide for the Care and Use of Laboratory Animals published by the US National Institutes of Health (NIH Publication No. 85–23, revised 1996). The rabbits were humanely sacrificed by injection of an overdose of sodium pentobarbital into the central ear vein (an approved Schedule 1 method). The heart was removed after confirmation of death of the rabbit.

### Development of a 3D anatomically-detailed model of the right atrium of the rabbit including the SAN and AVN

Our aim was the reconstruction of the right atrium, and the right atrium retained its shape by retaining other structures. The atria and a part of the ventricles were dissected for imaging. 3D images were obtained using high-resolution MR imaging carried out on a 11.7 T Bruker MR system (Bruker Medical, Ettlingen, Germany) at the University of Oxford. The voxel size after reconstruction was 26.4 µm×26.4 µm×24.4 µm (anisotropic). To form a convenient platform for numerical simulations, the anisotropic images were transformed into isotropic images with a voxel size of 30 µm×30 µm×30 µm and also 60 µm×60 µm×60 µm. MATLAB (version 7; The Math Works, Inc., Matick, MA, USA) was used to analyse the data: the anisotropic images were imported into the workspace of MATLAB and transformed into isotropic 3D images (voxel size, 30 µm×30 µm×30 µm; Figure S1A) using cubic interpolation.

The superior vena cava (SVC), crista terminalis (CT) and tricuspid valve (TV) could be recognised (Figure S1A). The 3D images were rotated to be in the normal anatomical orientation (x: right-left; y: ventral-dorsal; z: caudal-cranial) for segmentation. The right atrium structure was extracted based on the global threshold; the resulting binary images are shown in Figure S1B. Isosurface rendering was used to display the overall structure of the 3D model for visualisation (Figure S1C). Figure S1D shows the 3D model after segmentation, which is discussed below.

Segmentation was carried out semi-manually by analysing the MR imaging data from three directions (along x, y and z axes). First, the best orientation for segmentation was chosen. Secondly, the object of interest was extracted from the structure every 5∼10 images. Thirdly, interpolation was carried out to obtain the 3D object. Finally, the object was combined with other objects to produce a 3D multiple-object model. Eighteen objects were segmented in this study. The right atrial wall, crista terminalis, main branch (a thick muscle bundle projecting from the crista terminalis towards the atrial appendage), roof bundle (a thick muscle bundle projecting from the interatrial septum towards the atrial appendage), a small part of the right ventricle, the aorta with the aortic valve, the coronary sinus, the tricuspid valve, part of the mitral valve and the fossa ovalis were easy to recognise in the MR images and were segmented as described above.

**Figure 1:**
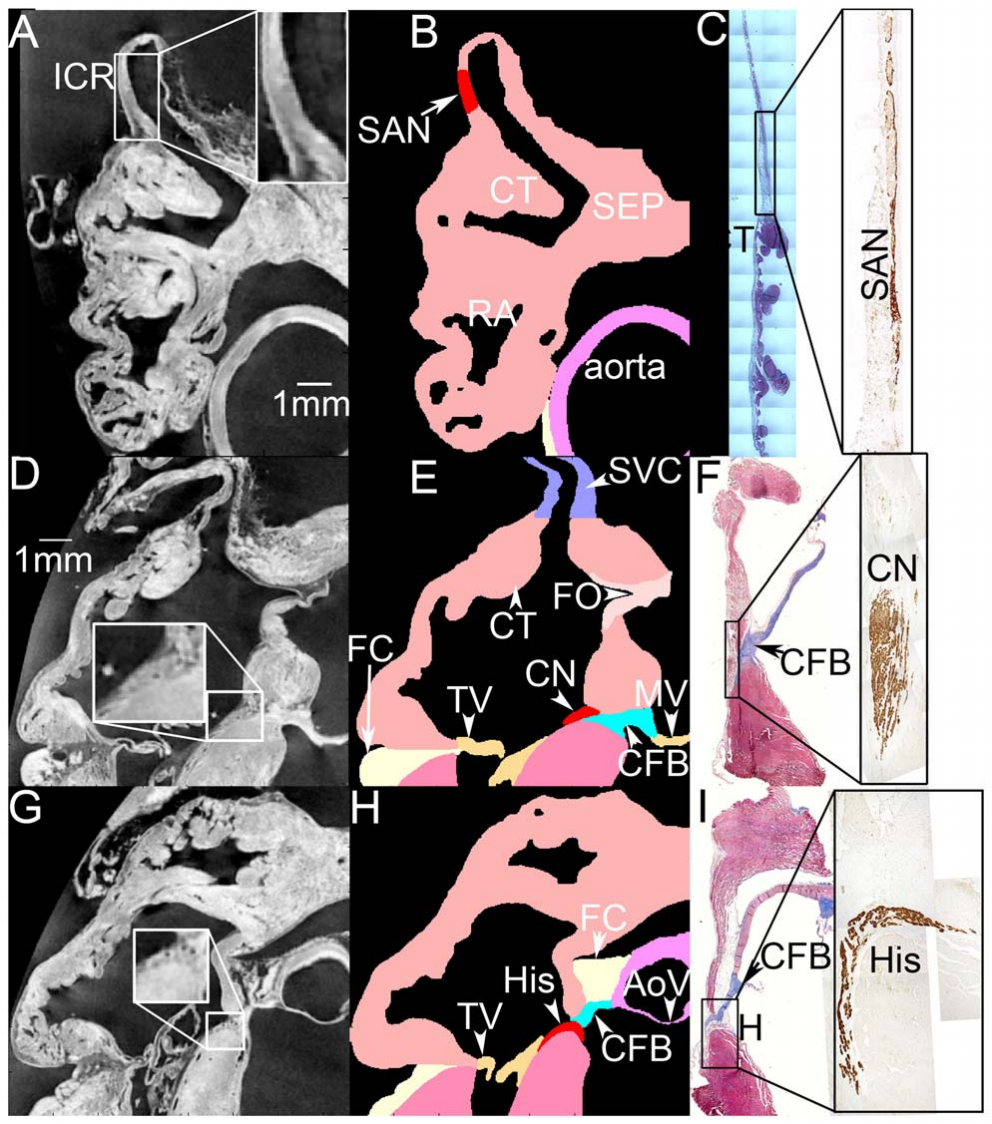
Identification of the SAN and AVN by comparing the MR images with Masson's trichrome stained and neurofilament-immunolabelled sections from the intercaval region and triangle of Koch region. A, D and G, MR images including the intercaval region (A), the compact node (part of the AVN; D) and the His bundle (G). Nodal regions are enlarged (boxes). B, E and H, corresponding segmented model sections including the SAN (B) and AVN (E and H). Different segmented structures are shown in different colours. C, F and I, sections through the SAN (C) and AVN (F and I) stained with Masson's trichrome and labelled for neurofilament (inset boxes). Masson's trichrome stains myocytes red and connective tissue blue. The neurofilament-positive (brown) cells are nodal. AoV, aortic valve; CFB, central fibrous body; CN, compact AVN; CT, crista terminalis; FC, outer fatty and connective tissue; FO, fossa ovalis; His, His bundle; ICR, intercaval region; MV, mitral valve; RA, right atrium; RV, right ventricle; SEP, interatrial septum; SVC, superior vena cava; TV, tricuspid valve.

The SAN and AVN were identified by comparing the MR images with Masson's trichrome stained and neurofilament-immunolabelled sections from the intercaval region (cut perpendicular to the crista terminalis [11]) and triangle of Koch (cut roughly perpendicular to septal leaflet of tricuspid valve [12]), as well as our previous 3D SAN and AVN models [11], [12], [58]. Neurofilament is a neuronal cytoskeletal protein that is exclusively expressed in the cardiac conduction system of the rabbit. The SAN and AVN can be recognised because they express neurofilament (brown label), whereas the surrounding atrial tissue does not (Figure 1C, F, I). To segment the SAN, MR images (Figure 1A) and Masson's trichrome stained (Figure 1C, left) and neurofilament-immunolabelled sections (Figure 1C, right) with the same features were compared. In the enlarged image within Figure 1A, it is possible to distinguish the SAN from the surrounding atrial tissue. To determine the extent of the SAN, the distance between the crista terminalis and the SAN centre and the length of the SAN centre as seen in Masson's trichrome stained and neurofilament-immunolabelled sections were measured (Figure 1C). Then the SAN was segmented accordingly. The result is shown in Figure 1B. The SAN periphery was not included in this model due to its complexity [59].

Figure 1D-F shows images through the compact part of the AVN. Once again, a MR image (Figure 1D) was compared with Masson's trichrome stained (Figure 1F, left) and neurofilament-immunolabelled (Figure 1F, right) sections with the same features. The neurofilament-positive (brown) cells in Figure 1F are AVN cells. In the enlarged image within Figure 1D, the AVN can be easily distinguished from the surrounding atrial muscle. The central fibrous body, stained blue with Masson's trichrome (Figure 1F, left), can be distinguished from the surrounding atrial muscle in the MR images by comparing Figure 1D with Figure 1F. Figure 1E shows a section from the segmented multiple-object model based on Figure 1D. Figure 1G-I shows comparable images through the His bundle; an analogous method was used to segment it (Figure 1H). The location of the tendon of Todaro and the right and left sinoatrial ring bundles were determined by comparing the MR images and Masson's trichrome stained sections of a similar region. It is well known that there is a conduction block zone from the SAN towards the interatrial septum [60]. It was not possible to detect the block zone in either MR images or tissue sections. Hence, the block zone was segmented according to previously published activation maps of the rabbit SAN [61].

**Figure 2:**
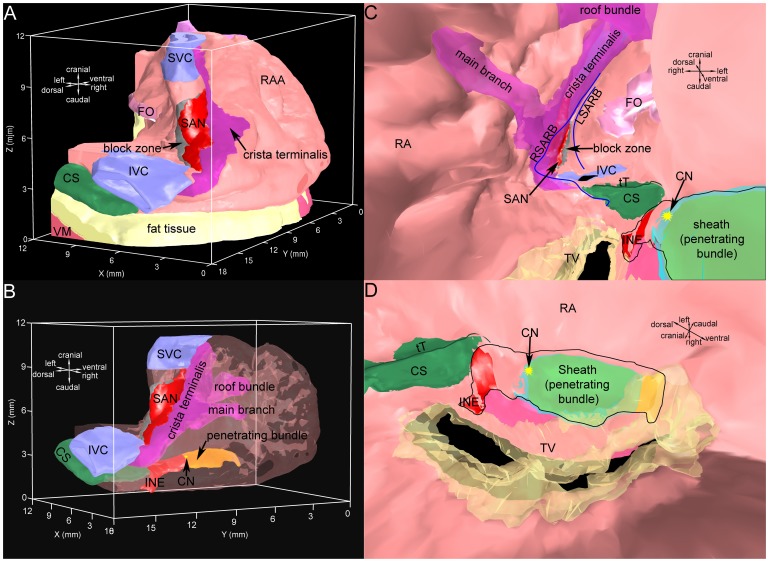
3D model of the rabbit right atrium including the SAN and AVN. A, dorsal oblique view of the model. B, model with transparent atrium to reveal internal structures. C, internal ventral view of the model. D, internal cranial right view of the model. Different segmented structures are shown in different colours. CN, compact node; CS, coronary sinus; INE, inferior nodal extension; IVC, inferior vena cava; FO, fossa ovalis; LSARB, left sinoatrial ring bundle; RAA, right atrial appendage; RSARB, right sinoatrial ring bundle; SVC, superior vena cava; tT, tendon of Todaro; TV, tricuspid valve.

Finally, a 3D right atrium volume model with ∼3.16 million elements and eighteen objects was constructed (Model S1). The objects are the right atrial wall, the crista terminalis, the main branch, the roof bundle, the SAN, the AVN (inferior nodal extension and penetrating bundle), part of the right ventricle, the aorta with the aortic valve, the superior vena cava, the inferior vena cava, the coronary sinus, the tricuspid valve, part of the mitral valve, the fossa ovalis, the central fibrous body, the block zone, and the outer fatty and connective tissue. The isosurface rendering technique was used to visualise the 3D volume model. The tendon of Todaro and the right and left sinoatrial ring bundles were highlighted using spline lines. Figure 2 shows various views of the 3D volume model. Figure 2A shows a dorsal oblique view of the model. The outer shape of the right atrium can be seen. The atrial muscle is shown in pink. The right atrial appendage can be easily recognised. The SAN (red) is located on the dorsal side within the intercaval region (the region between the superior vena cava and inferior vena cava). The crista terminalis (purple) is located on the right side of the SAN and the block zone (grey) is located on the left side of the SAN. The coronary sinus (green) runs under the inferior vena cava (blue), approximately perpendicular to the inferior vena cava. Figure 2B shows the model with transparent atrium to reveal internal structures. The ventricles, valves and central fibrous body were removed to reveal the AVN. The main branch (purple), roof bundle (purple) and AVN (inferior nodal extension, red; penetrating bundle, orange) can be seen through the transparent right atrium. Figure 2C is an internal ventral view. The tricuspid valve (yellow) and connective tissue sheath (blue) surrounding the penetrating bundle (orange) are transparent. The tendon of Todaro (green line) and the right and left sinoatrial ring bundles (dark blue lines) can be seen. The fossa ovalis (pink) is located on the left side of the left sinoatrial ring bundle. The SAN is surrounded by the right and left sinoatrial ring bundles. The three big muscle bundles (crista terminalis, main branch and roof bundle; purple) are clearly seen. The main branch branches from the crista terminalis. The roof bundle is analogous to a roof beam connecting the interatrial septum with the right atrial free wall. Figure 2D shows an internal cranial right view. The area surrounded by a thin black line is the AVN. Part of the inferior nodal extension (red) is covered by a thin layer of atrial tissue (pink). The penetrating bundle is covered by a connective tissue sheath (transparent blue). The ventral end of the penetrating bundle lies underneath the tricuspid valve (transparent yellow). The bright yellow stars in Figure 2B, C, D mark the position of the compact AVN.

### Simulation of the electrophysiological behaviour of the rabbit right atrium

#### Simulation domain

For numerical simulation, a simulation domain (Model S2) was defined to exclude some objects which are electrically inexcitable: aorta and aortic valve, tricuspid valve, mitral valve, central fibrous body and the fatty and connective tissue. Because the model did not include Purkinje fibres, the ventricle was not included in the simulation domain. It is not possible to separate the superior and inferior vena cava from the right atrium (Figure 1A, D) and, therefore, they were treated as excitable tissue with the same properties as atrial muscle. There are cardiac myocytes in the coronary sinus area [62] and again it was treated as excitable tissue with the same properties as atrial muscle. Cardiac myocyte orientation is important for the propagation of the action potential. It was not possible to detect myocyte orientation from the MR imaging data. However, it is reasonable to assume that cardiac myocytes run longitudinally along the muscle bundles of the atria. The pectinate muscle bundles within the right atrial wall are complex and difficult to segment. Also it is not possible to infer myocyte orientation within the intercaval region and interatrial septum by segmenting muscle bundles. Hence these parts of the atrial wall were defined as isotropic. Only three major muscle bundles (crista terminalis, main branch and roof bundle) were segmented and considered as anisotropic with myocytes running longitudinally along the muscle bundles. The SAN was simulated as an anisotropic material [60]. In summary, we have six zones (∼1.6 million elements) with different properties: part of the atrial wall (isotropic atrial tissue), three major muscle bundles (anisotropic atrial muscle), SAN (anisotropic), inferior nodal extension and penetrating bundle (isotropic material) and the block zone (passive atrial tissue – non excitable).

Because the right atrial model has structural complexity and consists of ∼1.6 million elements, instead of biophysically-detailed models, two caricature models of the action potential were used to simulate the action potential of the right atrium.

#### Electrophysiological models

The original FitzHugh-Nagumo model [56] was modified to simulate the spontaneous action potential of the SAN, which shows pacemaker behaviour:
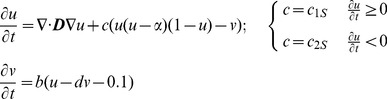
(1)where *u* and *v* are the excitation variable and recovery variable, **n** is a vector normal to the boundary, ***D*** is the diffusion tensor, and *α, b, c_1S_, c_2S_*, and *d* are parameters that define the shape of the excitation variable *u*. The action potential *V*_m_ and the threshold potential *V*_th_ are normalised by:
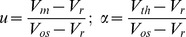
(2)where *V*_os_ is the overshoot potential and *V*_r_ is the resting potential.

The Rogers-modified FitzHugh-Nagumo model [63] is shown below:
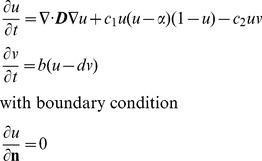
(3)


The first equation of the Rogers-modified FitzHugh-Nagumo model (above) was modified further to simulate the action potential of the atrial muscle, inferior nodal extension and penetrating bundle:
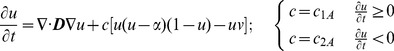
(4)


In this equation, *c*_1A_ and *c*_2A_ are different from *c*_1_ and *c*_2_ of Equation 3.

The SAN periphery was not included in the 3D atrial model because of its complexity [11]. However, a border area of the SAN, 0.24 mm (4 elements) wide, was defined as an ideal SAN periphery using Equation 1. Its property changes gradually as follows:
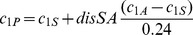
(5)where *c*_1P_ is *c*_1S_ in the SAN model (Equation 1). *dis*SA is the minimum distance between one SAN element and atrial elements.

The block zone was modelled as a passive tissue:
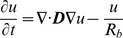
(6)where *R_b_* is resistivity. *R_b_* was set to 0.5, which is sufficient to inhibit the action potential.

**Figure 3:**
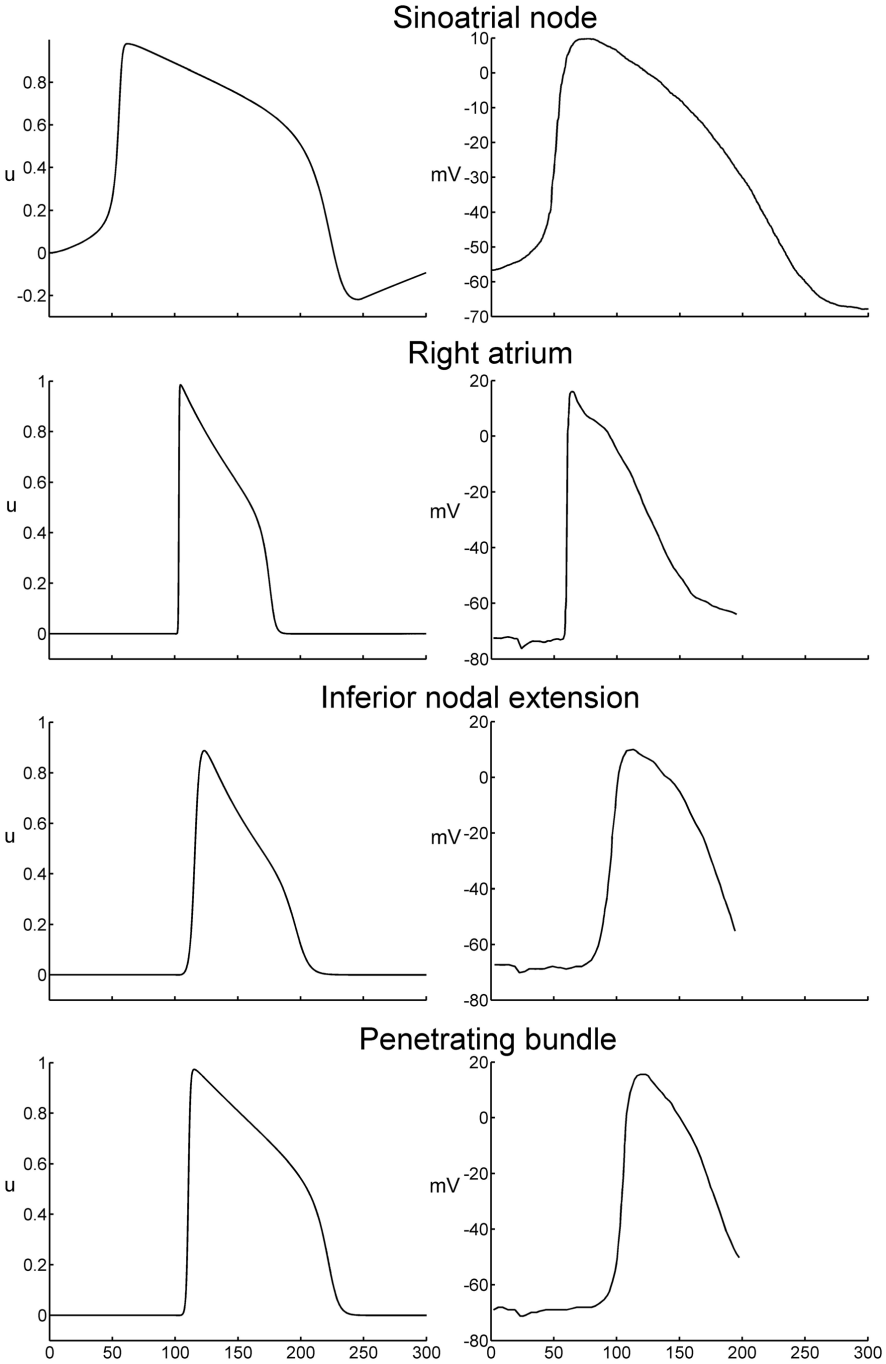
Comparison of action potential waveforms in the SAN, right atrium, inferior nodal extension and penetrating bundle in simulations (left) and experiment (right). Experimental recordings from Kodama et al. [95] (SAN) and de Carvalho and de Almeida [96].

To define model parameters for SAN, atrial muscle, inferior nodal extension and penetrating bundle, a 50×5×5 elements strand tissue model was used [64]. Stimulation was applied to the first three layers of elements (3×5×5). The conduction velocity was measured as the average conduction velocity calculated from the 10^th^ element layer to the 40^th^ element layer. Action potential duration was measured at 90% repolarization. A standard *S1–S2* protocol was used to measure the refractory period. The spontaneous cycle length of the SAN (set by the modified Fitzhugh-Nagumo model) is 330 ms (corresponding to a heart rate of 182 beats/min). Table 1 lists the parameters for the SAN, atrial muscle, inferior nodal extension and penetrating bundle. Table 2 lists the conduction velocity, maximum upstroke velocity, action potential duration and refractory period of the SAN, atrial muscle, inferior nodal extension and penetrating bundle from simulations and compares them to experimentally determined values. The data show that the majority of simulation results are within the range of experimental results. Figure 3 shows action potential waveforms from simulations (left) and experiment (right). The simulated action potential waveforms are a reasonable fit to the experimental waveforms.

**Table 1:** Parameters for the Fitzhugh-Nagumo model for the SAN, atrial muscle and AVN.

Parameters	SAN	Atrial muscle	Inferior nodal extension	Penetrating bundle
***D***	2	7	2	2
*c_1_*	1	12.7	1.45	3.05
*c*_2_	0.22	1.84	1	1
*b*	0.003	0.01	0.013	0.0048
*d*	3.5	2.475	2.5	2

**Table 2:** Measurements of conduction velocity, maximum upstroke velocity of the action potential, action potential duration and refractory period of the SAN, atrial muscle and AVN from the Fitzhugh-Nagumo model and experiment.

		SAN	Atrial muscle	Inferior nodal extension	Penetrating bundle
**Conduction velocity (m/s)**	**simulation**	0.0673	0.5333	0.0949	0.1413
	**experiment**	0.02–0.12 [65]	0.3–0.8 [81]	0.02–0.1 [82]	0.1–0.15
**Maximum upstroke velocity (ms^−1^)**	**Simulation**	0.124	1.583	0.1578	0.3755
	**experiment**	0.075–0.16 [83]–[88]	1.5±0.5 [89]	0.164 [39]	0.376 [39]
**Action potential duration (ms)**	**Simulation**	185	75.05	93.7	118.07
	**experiment**	107–151 [88], [90], [91]	77±5 [89]	97.2 [39]	117.5 [39]
**Refractory period (ms)**	**Simulation**	283	82	91	154
	**experiment**	166±30 [92]	68±11 [92] 81±5 [39]	91±10 [93] 91±12 [39] 100±9 [94]	−

The maximum upstroke velocity is expressed as 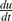, where *u* is the excitation variable and *t* is time. For the experimental data, 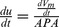, where *V*_m_ is the membrane potential and *APA* is action potential amplitude.

The SAN and three major muscle bundles (crista terminalis, main branch and roof bundle) were considered as anisotropic. In the SAN, cells are reported to be oriented in different directions (forming a mesh) [11]. Experimental results suggest that the SAN is anisotropic, and the conduction velocities near the centre of SAN towards the SVC and IVC are faster than towards the crista terminalis and interatrial septum [65]–[67]. Hence it is reasonable to assume that most myocytes in the SAN run parallel to the crista terminalis. Let ***f*** denote the fibre orientation: ***f*** = *l* ***i***+*m* ***j***+*n* ***k***, where ***i***, ***j*** and ***k*** are unit vectors of a Cartesian coordinate system; *l*, *m* and *n* are direction cosines, respectively. The properties of anisotropic fibres are introduced through an anisotropic diffusion tensor ***D*** *_f_* in the fibre coordinate system:
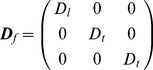
(7)where *D_l_* is the diffusion in the fibre direction and *D_t_* is the diffusion in the transverse direction. Then it is transformed to the global coordinate system as
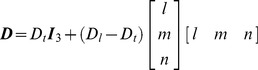
(8)where ***I_3_*** is the identity matrix. The diffusion anisotropy ratio was defined as 10 (*D_l_/D_t_*). This yields a ∼3.2 conduction velocity anisotropy ratio since conduction velocity is proportional to the square root of the diffusion [68].

#### Cellular automaton model

**Figure 4:**
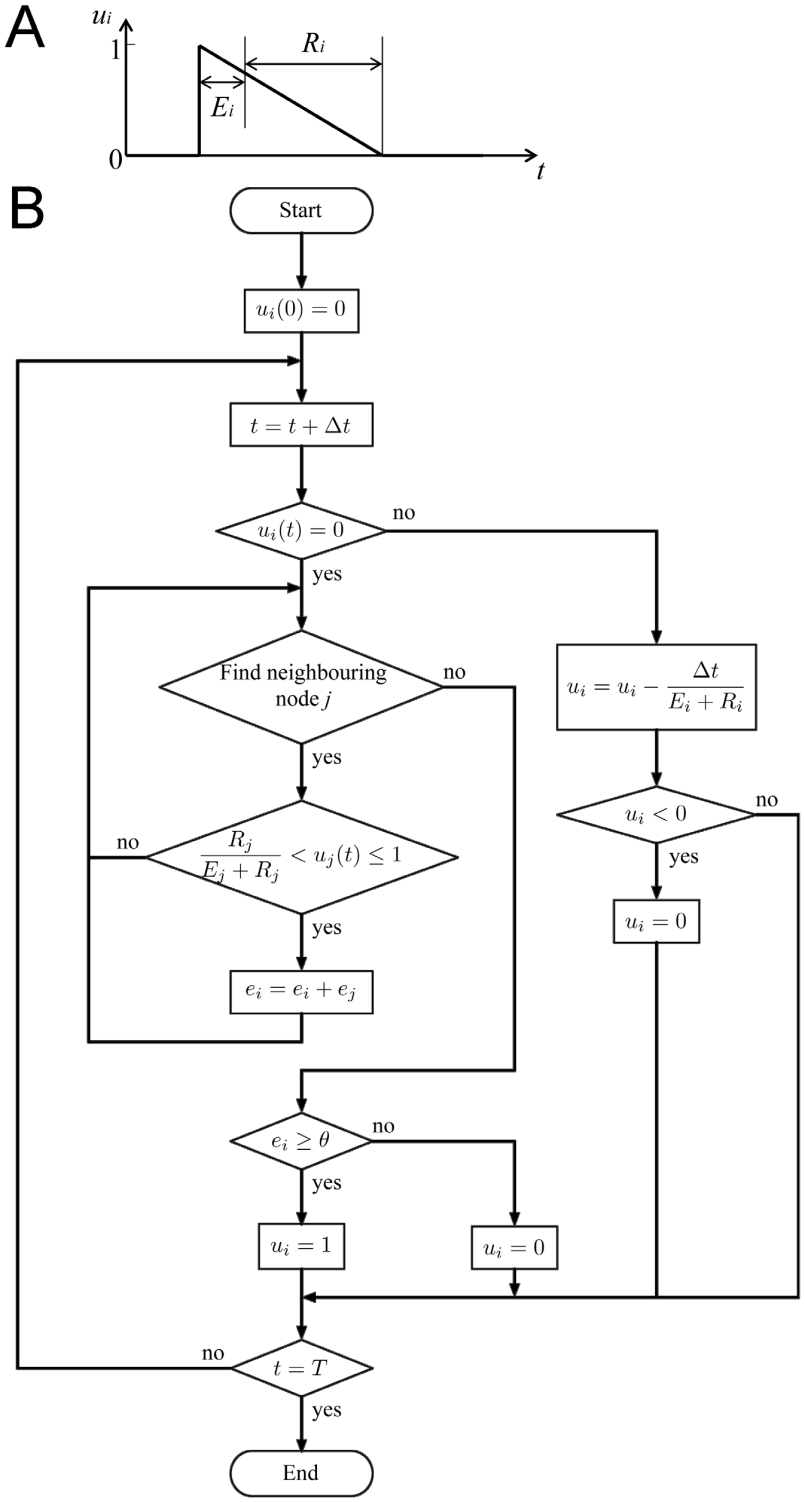
Cellular automaton model. A, time course of state (*u*) of node *i* in the cellular automaton model. If node *i* is excited the node state *u_i_* is changed from 0 (resting) to 1 (excited). *u_i_* then declines back to 0. B, program flow-chart of the cellular automaton model. *E_i_*, the excited period; *R_i_*, the refractory period. *t*, time; Δ*t*, the time increment; *T*, the end time of simulation; *e_i_*, the excitation counter; *θ*, the threshold.

In the cellular automaton model, each node can be in one of three states: resting, excited or refractory. In the model, each node *i* has a variable (*u*_i_), which signifies node state. When the node state is resting, *u*_i_ is set to 0. Once a node is excited, the node state is changed to 1. After that the node state is gradually decreased from 1 to 0. The speed of decrease determines the refractory period. The ‘action potential’ in the cellular automaton model is, therefore, characterised by the time at which the node is excited and at which it returns to the resting state, and the shape of the action potential can be considered triangular (Figure 4A). In the simulation program, if a node is in the resting state, the program checks at every time step whether one of its neighbours *j* is in the excited state. If so, an inner variable (*e_i_*) called the excitation counter (corresponding to ionic current from each excited neighbouring myocyte) is increased at each time step. If the excitation counter exceeds a predefined threshold (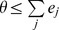), the node switches to the excited state (*u*_i_ = 1). In the excited state, a node can excite its neighbours for predefined time steps (*E_i_*) and then switches to the refractory state, where it again stays for a constant number of time steps (*R_i_*). At the end of the refractory state, the node switches back into the resting state (*u*_i_ = 0). Figure 4B shows a flow-chart of the program. Changes in conduction velocity and refractory period in different tissues (shown in Table 3) were introduced as discussed by Li et al. [12].

**Table 3:** Measurements of conduction velocity and refractory period of atrial muscle and AVN from the cellular automaton model and experiment.

		Atrial muscle	Inferior nodal extension	Transitional zone	Penetrating bundle
**Conduction velocity (m/s)**	**simulation**	0.27–0.55	0.05–0.114	0.154	0.104
	**experiment**	0.3–0.8 [81]	0.02–0.1 [82]	0.35±0.17 [70]	0.1–0.15
**Refractory period (ms)**	**simulation**	81	94	134	154
	**experiment**	68±11 [92] 81±5 [39]	91±10 [93] 91±12 [39] 100±9 [94]	127±9 [39] 141±15 [93]	_

All simulations were carried out on a high-performance computing cluster which has 10 quad-processors nodes.

## Results

### Conduction of the action potential from the SAN to the AVN during sinus rhythm

**Figure 5:**
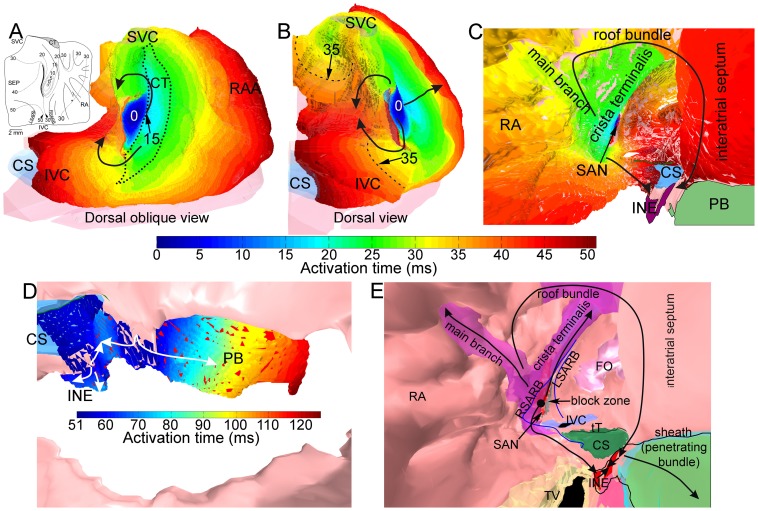
The sequence of right atrial activation during normal sinus rhythm in the 3D model of the rabbit right atrium. A–E, external (A, dorsal oblique view and B, dorsal view) and internal (C–E) views of the model. In A, the crista terminalis is outlined by a dotted line. In B, the dashed line is an example isochrone at 35 ms. In A to D, the activation sequence is shown by a colour scale and the arrows show the direction of action potential conduction. In E, different segmented structures are shown in different colours and the arrows summarise action potential conduction from the SAN to the AVN. CS, coronary sinus; INE, inferior nodal extension; IVC, inferior vena cava; FO, fossa ovalis; LSARB, left sinoatrial ring bundle; PB, penetrating bundle; RA, right atrium; RAA, right atrial appendage; RSARB, right sinoatrial ring bundle; SVC, superior vena cava; TV, tricuspid valve.

**Figure 6:**
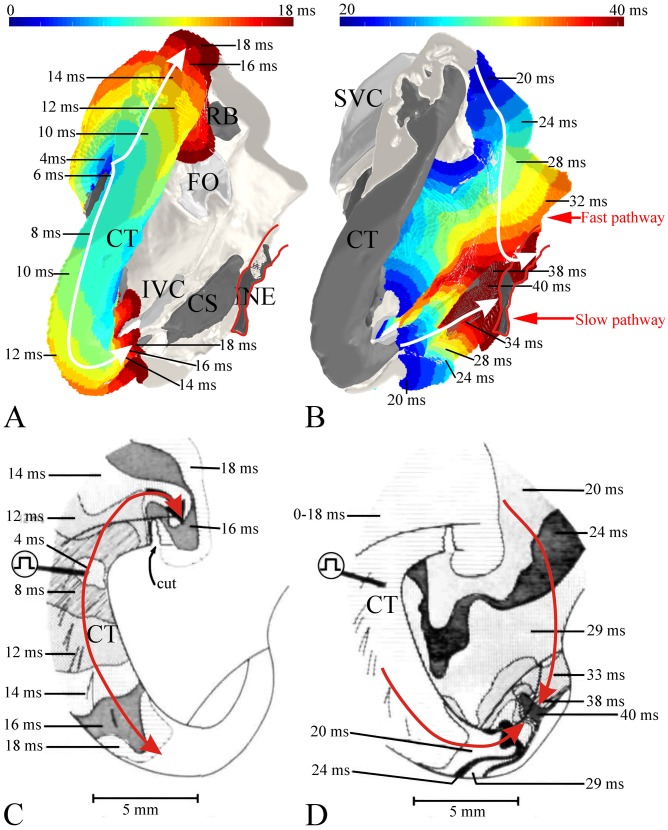
Action potential conduction from the SAN to the AVN. A and B, simulation of action potential conduction from the SAN to the AVN in the model of the right atrium during sinus rhythm (A, early times; B, later times). Internal right view of the model shown. Anatomical structures are shown in grey scale. The activation sequence is shown by a colour scale and the arrows show the direction of action potential conduction. Activation times are relative to the arrival of the action potential at the crista terminalis from the SAN. C and D, equivalent experimental data for the rabbit right atrium from Spach et al. [72]. In the experiment, the crista terminalis was stimulated close to the site where the action potential is expected to arrive first from the SAN. CN, compact node; CS, coronary sinus; CT, crista terminalis; FO, fossa ovalis; INE, inferior nodal extension; IVC, inferior vena cava; PB, penetrating bundle; RB, roof bundle; SVC, superior vena cava.

The conduction of the action potential through the right atrium during sinus rhythm was simulated using the 3D anatomically-detailed right atrium model, together with a set of Rogers modified FitzHugh-Nagumo models. Figure 5 shows the activation sequence of the right atrium during sinus rhythm. The action potential was generated spontaneously in the centre of the SAN at 0 ms (Figure 5A, B). It spread preferentially in an oblique cranial direction (as a result of the orientation of nodal myocytes) and reached the crista terminalis first at ∼15 ms and then spread in a radial fashion to the rest of the right atrium (Figure 5A, B). It had to propagate around the block zone to reach the interatrial septum at ∼35 ms (Figure 5A, B). In the model, the action potential reached the rest of the right atrium in ∼50 ms (Figure 5A, B). Propagation from the SAN to the AVN is shown in Figure 6A, B: the action potential rapidly spread down the crista terminalis (Figure 6A) and it entered the AVN at the inferior nodal extension ∼40 ms after it first entered the crista terminalis (Figure 6B) and ∼50 ms after its initiation in the SAN (Figure 5C). Meanwhile, the action potential also spread up the crista terminalis (Figure 6A) and down the interatrial septum and it entered the AVN via the compact node at the junction of the inferior nodal extension with the penetrating bundle again ∼40 ms after it first entered the crista terminalis (Figure 6B) and ∼50 ms after its initiation in the SAN (Figure 5C). This suggests that there are two principal pathways from the SAN to the AVN: one via the crista terminalis and the second via the interatrial septum. Although the action potential arrived at the AVN via both pathways at approximately the same time, the action potential arriving via the crista terminalis entered the atrioventricular conduction pathway at an upstream site, whereas the action potential arriving via the interatrial septum entered at a downstream site. Once the action potential from the crista terminalis had entered the AVN, it then began propagating along the inferior nodal extension towards the penetrating bundle (Figure 5D). Once the action potential from the interatrial septum had entered the AVN, the action potential propagated anterogradely along the penetrating bundle (Figure 5D). However, it also propagated retrogradely along the inferior nodal extension (Figure 5D) and finally collided with the action potential coming from the opposite direction (Figure 5D). Figure 5E is a summary of the activation sequence from the SAN to the AVN. The simulation suggests that the most important pathway for the action potential from the SAN to the AVN is the interatrial septum; the pathway from the SAN to the AVN via the crista terminalis is secondary. The action potential propagated anterogradely along the penetrating bundle and it reached the bundle of His at ∼120 ms after its initiation in the SAN (Figure 5D).

### Nodal activity during atrial fibrillation

**Figure 7:**
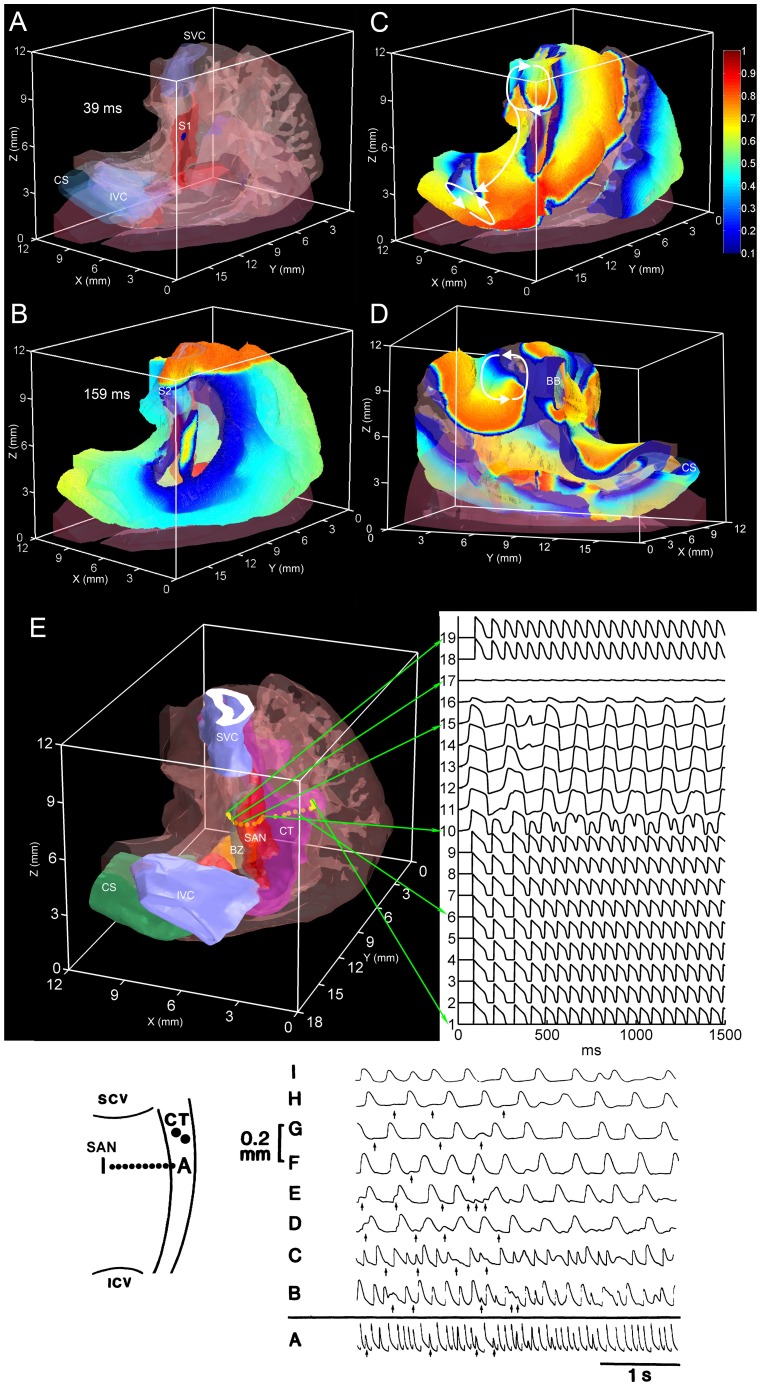
Behaviour of the SAN during atrial fibrillation. Panels A–E show external views (A–C and E, dorsal oblique view; D, ventral oblique view) of the model of the right atrium with transparent atrial muscle and different segmented structures shown in different colours. A–D, snapshots of the action potential distribution on the epicardial surface on initiation of an *S1* stimulus (A), on initiation of an *S2* stimulus, and at two times points after the induction of atrial fibrillation (C and D). The reentry loops are highlighted by arrows. E, right atrium model showing the location of the recording sites of the action potentials shown on the right. The sites lie along a line perpendicular to the crista terminalis through the SAN. Inset, experimental data showing the behaviour of the rabbit SAN during atrial fibrillation. Left, schematic diagram of the preparation showing the location of the recording sites of the action potentials on the right. The sites lie along a line perpendicular to the crista terminalis through the SAN. From Kirchhof et al. [75]. BB, bachmann bundle; BZ, block zone; CS, coronary sinus; CT, crista terminalis; IVC (or ICV), inferior vena cava; SVC (or SCV), superior vena cava.

In the model, an *S1–S2* protocol was used to simulate an atrial fibrillation-like arrhythmia. The *S1* ‘stimulus’ was a normal sinus rhythm beat initiated in the centre of SAN (Figure 7A; 39 ms). The activation time was counted from starting the simulation. The *S2* stimulus was a premature planar stimulation applied to the superior vena cava (Figure 7B; 159 ms). Three reentrant circuits were observed (Movie S1, S2 and S3). The first reentrant circuit was located on the SVC and was a sustained reentrant circuit (Figure 7C; top). The second reentrant circuit was located on the IVC; it started at 495 ms and it did not stop by the end of the simulation at 1500 ms (Figure 7C; bottom). The third reentrant circuit was located on the atrial free wall and started at 780 ms and stopped at 1020 ms (Figure 7D). Figure S2 shows a set of snapshots of the action potential distribution on the epicardial surface during the atrial reentrant arrhythmia. Figure S3 shows a set of snapshots of the action potential distribution on the endocardial surface during the atrial reentrant arrhythmia.

Figure 7E shows the predicted behaviour of the SAN during atrial fibrillation. It shows action potentials recorded at different sites along a line perpendicular to the crista terminalis through the SAN: atrial muscle (e.g. site 1), SAN periphery (e.g. site 10), SAN centre (e.g. site 15), block zone (sites 16 and 17) and interatrial septum (sites 18 and 19). There is a high frequency of fibrillatory action potentials in the atrial muscle (∼14 Hz; e.g. site 1), chaotic activity in the periphery of the SAN (site 10) and a slow frequency of the action potentials in the centre of the SAN (∼6 Hz; e.g. site 15) as a result of alternating 2∶1 and 3∶1 SAN entrance block (compare action potentials in and around the periphery of the SAN at sites 9, 10 and 11). The model predicts that the SAN is protected against the high rate of fibrillatory action potentials and is not overdriven - this behaviour is due to the long refractory period of the SAN (Table 2).

**Figure 8:**
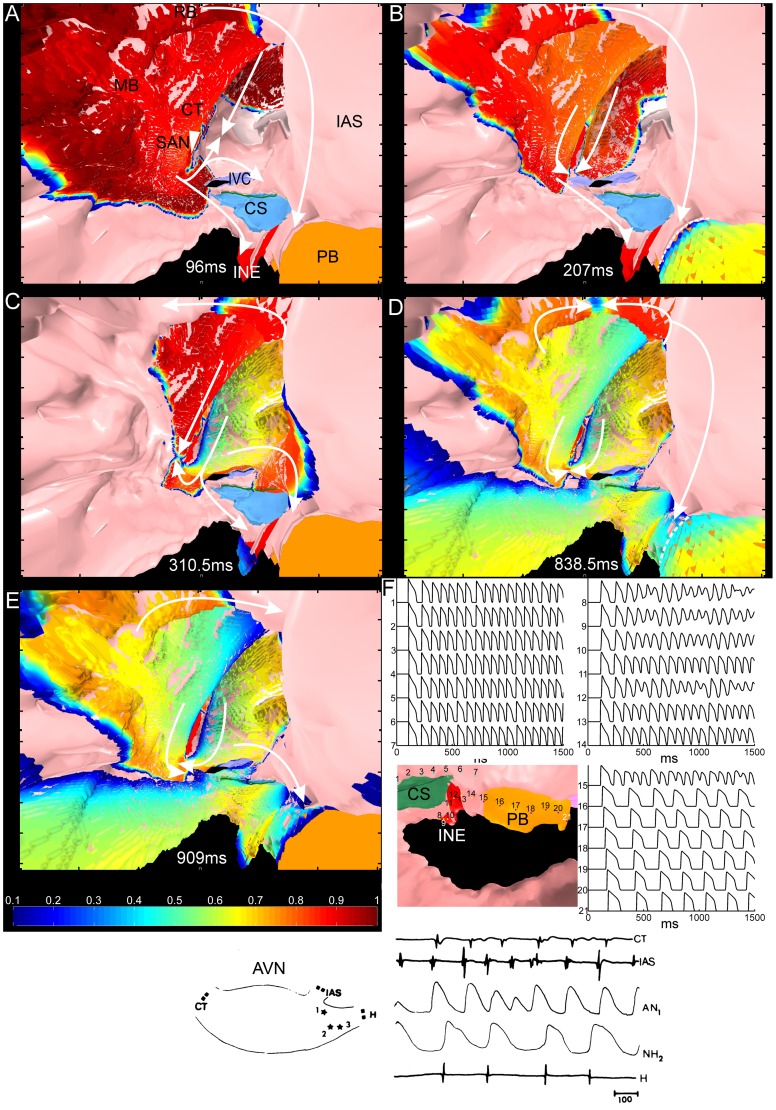
Behaviour of the AVN during atrial fibrillation. Panels A–E show a ventral internal view of the model of the right atrium with different segmented structures shown in different colours. A–E, snapshots of the action potential distribution on the endocardial surface during normal sinus rhythm (A) and at different times points after the induction of atrial fibrillation (B–E). The white arrows highlight wave propagation. The white dotted line in B and D indicates a line of conduction block. F, right atrium model showing the location of the recording sites of the action potentials shown above and on the right. Inset, experimental data showing the behaviour of the rabbit AVN during atrial fibrillation. Left, schematic diagram of the preparation showing the location of the recording sites of the electrograms and action potentials on the right. From Mazgalev et al. [80]. AN, atrionodal cell; CS, coronary sinus; CT, crista terminalis; IAS, interatrial septum; INE, inferior nodal extension; IVC, inferior vena cava; H, His bundle; MB, main branch; NH, nodal-His cell; PB, penetrating bundle; RB, roof bundle.

Figure 8 shows the predicted behaviour of the AVN during atrial fibrillation. Figure 8A–E shows an internal view of the right atrium and atrial action potential propagation during atrial fibrillation. Action potential propagation during normal sinus rhythm (*S1*) is shown in Figure 8A. Figure 8B–E shows action potential propagation at different time points of the atrial fibrillation. During atrial fibrillation, the action potential entered the AVN at different times through both pathways (fast and slow). Some reentrant action potentials reached the fast pathway and the compact node from the roof bundle and the interatrial septum (Figure 8B, D), whereas others reached the fast pathway from the area of inferior vena cava and coronary sinus (Figure 8C, E). Some reentrant action potentials were blocked (dashed lines in Figure 8B, D), because of the long refractory period of the penetrating bundle (Table 2). Figure 8F shows action potentials recorded at the atrioventricular junction axis: atrial muscle (sites 1–7), inferior nodal extension (sites 8–14) and penetrating bundle (sites 15–21). It shows the high frequency (∼14 Hz) fibrillatory action potentials in the atrial muscle (sites 1–7), chaotic activity in some transitional regions of the AVN (e.g. site 15) and a low frequency (∼5 Hz) of action potentials in the distal penetrating bundle (site 16), as the result of ∼3∶1 Wenckebach block (compare recordings at sites 15 and 16). The Wenckebach block is the result of the long refractory period of the AVN (Table 2). In summary, the simulation shows that the high frequency fibrillatory activity is filtered by the AVN; this is important, because it prevents the ventricles from being paced too fast.

### Simulation of AVN reentry and a ventricular echo beat generated by premature ventricular stimulation

**Figure 9:**
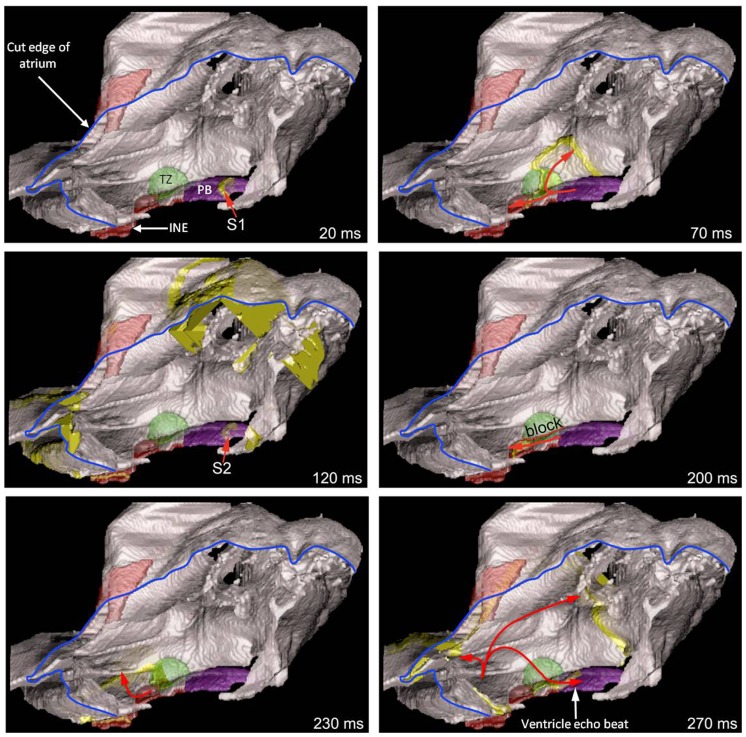
Simulation of a ventricular echo beat. A right view of the model of the right atrium is shown with one face of the right atrium removed (the blue line shows the cut edge of the right atrium). Different segmented structures are shown in different colours. The panels show snapshots of activation after *S1–S2* stimulation of the penetrating bundle. The thick yellow lines show the advancing wavefront of activation. The arrows show the direction of action potential conduction. 20 ms, retrograde conduction of the action potential along the penetrating bundle following *S1* stimulation. 70 ms, retrograde conduction of the action potential along the fast (transitional zone; green) and slow (inferior nodal extension, red) pathways out of the AVN following *S1* stimulation. 120 ms, retrograde conduction of the action potential along the penetrating bundle following *S2* stimulation. Note the action potential following *S1* stimulation has reached the outer parts of the right atrium. 200 ms, block of conduction along the fast pathway (line of block, red dotted line) and retrograde conduction along the slow pathway following *S2* stimulation. 230 ms, retrograde conduction out of the slow pathway and into the right atrium following *S2* stimulation. 270 ms, retrograde conduction throughout the right atrium and reentry into the AVN – the action potential conducted anterogradely through the transitional zone to the penetrating bundle. It is this action potential that would result in a ventricular echo beat. INE, inferior nodal extension; PB, penetrating bundle; TZ, transitional zone.

A premature ventricular action potential can elicit a ventricular echo beat as a result of retrograde conduction through the AVN followed by AVN reentry and anterograde conduction through the AVN [69]. This was investigated using the 3D anatomically-detailed right atrium model together with the cellular automaton model of the action potential (less computationally intensive than the Fitzhugh-Nagumo model). AVN reentry is dependent on the fast pathway (a transitional zone contacting the compact node) and the slow pathway (inferior nodal extension) into the AVN having different refractory periods; the refractory period of the fast pathway is longer than that of the slow pathway (Table 3) [39], [70]. The model of the right atrium (Figure 2) does not have a transitional zone, because it was not detectable in the MR images. Therefore, a transitional zone (shown in green in Figure 9) was segmented based on our previous 3D rabbit AVN model [12]. To replicate ventricular activation, a *S1* stimulus was applied at the penetrating bundle of the AVN (Figure 9; 20 ms time point) and then a *S2* stimulus was applied with a short cycle length (Figure 9; 120 ms time point). The *S1* action potential was conducted retrogradely from the penetrating bundle to the atrium via both fast and slow pathways, i.e. by both the transitional zone and the inferior nodal extension (Figure 9; 70 ms time point). After applying the *S2* stimulus, conduction was blocked through the transitional zone (Figure 9; 200 ms time point), because of its long refractory period (Table 3). However, the refractory period of the inferior nodal extension is shorter (Table 3) and the action potential was conducted to the atrium via the inferior nodal extension (Figure 9; 230 ms time point). From the atrium, the action potential was then able to enter the transitional zone, because its refractory period had ended (Figure 9; 270 ms time point). From the transitional zone, the action potential was then conducted anterogradely to the penetrating bundle (Figure 9; 270 ms time point); in a heart, this would result in a ventricular echo beat.

## Discussion

In this study, a 3D anatomically-detailed rabbit right atrium model including the two nodes was generated. Using this anatomical model together with mathematical caricature models of the action potential, we were able to replicate some known nodal electrophysiology: (i) the two routes of action potential conduction from the SAN into the AVN via the crista terminalis and interatrial septum; (ii) the dominance of the interatrial septum route over the crista terminalis route; (iii) little or no overdrive of the SAN during atrial fibrillation; (iv) reduction of action potential frequency by the AVN during atrial fibrillation; and (v) ventricular echo beats. The success of the simulations is a validation of the model. It also demonstrates that the phenomena can be explained on the basis of the elements incorporated in the model and in particular on anatomy, conduction velocities and refractory periods.

### Comparison of model and experiments

#### Conduction of the action potential from the SAN to the AVN during sinus rhythm

In the simulation of sinus rhythm, the action potential from the SAN had to propagate around the block zone to reach the interatrial septum at ∼35 ms (Figure 5A, B). A similar pattern is seen in experiments [60]. As an example, the inset in Figure 5A shows conduction from the leading pacemaker site in the rabbit SAN – the action potential spread preferentially in an oblique cranial direction, reaching the crista terminalis in 15–20 ms, and propagated around the block zone to reach the interatrial septum at 40–50 ms. In the model, the action potential reached the rest of the right atrium in ∼50 ms (Figure 5A, B). There is little experimental data to compare this too. De Carvalho et al. [71] worked with a large right atrial preparation from the rabbit including both the SAN and AVN and the furthest reaches of the preparation were activated in 70–80 ms. The model suggests that there are two routes for the action potential from the SAN to the AVN: the crista terminalis and the interatrial septum (Figure 6A, B). This is consistent with experimental data from the rabbit from Spach et al. [72] shown in Figure 6C, D. In this experimental study, the crista terminalis was stimulated (at 0 ms) close to the leading pacemaker site in the SAN. As in the equivalent simulation (Figure 6A, B), the action potential rapidly spread down the crista terminalis and entered the AVN (inferior nodal extension) as well as up the crista terminalis and down the interatrial septum where it entered the AVN downstream of the inferior nodal extension. In the work of Spach et al. [72] the action potential entered the AVN at ∼40 ms after stimulation of the crista terminalis (Figure 6C, D). In other work on the rabbit [71], the action potential entered the AVN at ∼50 ms after its initiation in the sinus node. These timings are similar in the corresponding simulations (Figures 5C and 6A, B). Both in the human and animal models, it is well known that there are dual inputs into the AVN: the slow and fast pathways [73]. The slow pathway is the inferior nodal extension and the fast pathway is the transitional zone leading into the compact node [12]. The simulations show that the crista terminalis connects to the slow pathway, i.e. the inferior nodal extension, whereas the interatrial septum connects to the fast pathway, i.e. the region of the transitional zone and compact node (Figure 5E). The simulations suggest that the most important pathway for the action potential from the SAN to the AVN is the interatrial septum followed by the fast pathway; this is because the action potential enters the atrioventricular conduction axis at a more downstream site via this route (shortening the conduction time to the His bundle). This is consistent with what is known: both in the human and animal models, it is well known that the fast pathway is the primary pathway for atrioventricular conduction [73]. The slow pathway only operates if the fast pathway fails (for example, during premature stimulation when the fast pathway fails because of its long refractory period [39]). In the model, the action potential propagated anterogradely along the penetrating bundle and reached the bundle of His at ∼120 ms (Figure 5D). This is less than an experimental value of 140 ms for the rabbit heart from De Carvalho et al. [71]. However, the PR interval is the time from atrial depolarization to the first activation of the ventricular conduction system and in the rabbit is 62 ms (Boyett, unpublished data). If the time from initiation of the action potential in the SAN to the arrival of the action potential in the atria is 15 ms (Figure 5A), then the time from the initiation of the action potential in the SAN to the first activation of the ventricular conduction system is ∼80 ms. In summary, there is good agreement between model and experiment in terms of conduction of the action potential from the SAN to the AVN. The success of the simulations indicates that the principal factor determining the pattern of conduction from the SAN to the AVN is anatomy, in particular the arrangement of fast conducting atrial muscle bundles with longitudinally arranged myocytes, although the slow conduction velocity of the inferior nodal extension is another contributory factor (working against the crista terminalis-slow pathway route).

From simulations, conduction velocities in the SAN were measured as 0.028 m/s in the transverse direction and 0.089 m/s in the longitudinal direction; conduction velocities in the crista terminalis were measured as 0.183 m/s in the transverse direction and 0.417 m/s in the longitudinal direction. The conduction velocity anisotropy ratio in the SAN (∼3.16) is within the range of experimental results (1.9∼4.1) from rabbit SAN preparations [65]. The conduction velocity anisotropy ratio in the crista terminalis (∼2.28) is similar to that measured experimentally in rabbit crista terminalis preparations (1.7∼4.1 depending on age [74]).

#### Nodal activity during atrial fibrillation

The rabbit heart has been widely used in the study of atrial fibrillation [75]–[78]. In the simulation of atrial fibrillation (Figure 7), the fibrillation frequency was 14 Hz in the atrial muscle. This is similar to that measured in the rabbit experimentally under baseline conditions (11.6 Hz [75]; 5.9–6.5 Hz [78]) and following vagal stimulation (12–13 Hz [78]). In the model, the arrhythmia was maintained by reentry located at the SVC and IVC. This is consistent with observations from patients with paroxysmal atrial fibrillation [79]. Kirchhof et al. [75] studied the SAN during atrial fibrillation in the rabbit and an example from their work is shown in the inset at the bottom of Figure 7; it shows action potentials recorded at different sites from the atrial muscle (site A), SAN periphery (e.g. site B) and SAN centre (e.g. site I). The behaviour is qualitatively similar to that predicted by the model: there is a high frequency of fibrillatory action potentials in the atrial muscle (site A), chaotic activity in the periphery of the SAN (e.g. site B) and a slow frequency of the action potentials in the centre of the SAN (e.g. site I) as a result of high degree (5∶1) SAN entrance block (compare action potentials at sites A–D). Mazgalev et al. [80] studied the AVN during atrial fibrillation in the rabbit and an example from their work is shown in the inset at the bottom of Figure 8; it shows extracellular electrograms recorded from the crista terminalis (CT), interatrial septum (IAS) and His bundle (H) as well as intracellular action potentials recorded at upstream (site 1) and downstream (site 2) sites in the atrioventricular conduction axis. Again the behaviour is qualitatively similar to that predicted by the model: there is a high frequency of fibrillatory action potentials in the atrial muscle (CT and IAS), chaotic activity in a transitional region (site 1) and a slow frequency of action potentials downstream of this, including in the His bundle (corresponding to ∼2∶1 Wenckebach block). In summary, there is good agreement between model and experiment in terms of the behaviour of the nodes during atrial fibrillation. The success of the simulations can be attributed to the long refractory period of the nodal tissues.

#### AVN reentry and ventricular echo beats

A premature ventricular action potential can elicit a ventricular echo beat as a result of AVN reentry [69] and the model was able to successfully simulate this (Figure 9).

### Limitations of the study

It was not possible to obtain fibre orientation from the MR images and only three major muscle bundles (crista terminalis, main branch and roof bundle) were segmented in order to introduce the well known anisotropic property of the bundles. The SAN was also assumed to be anisotropic in order to replicate the known anisotropic conduction in the SAN, whereas the rest of the right atrium was assumed to be isotropic. Although the success of the simulations demonstrates that the level of detail in the model is sufficient to explain the various behaviours, incorporation of fibre orientation throughout the right atrium model is expected to improve the accuracy of simulations. Mathematical caricature models of the action potential were used to make computation more tractable. Once again, although the success of the simulations demonstrates that the level of detail in the models is sufficient to explain the various behaviours, use of biophysically-detailed models of the action potential is expected to improve the accuracy of simulations.

## Supporting Information

Figure S1 **3D segmentation.** A, isotropic 3D MR images; B, converted 3D binary images; C, the 3D model before segmentation; D, the 3D model after segmentation. CT, crista terminalis; RA, right atrium; RV, right ventricle; SAN, sinoatrial node; SVC, superior vena cava; TV, tricuspid valve.
(TIF)

Figure S2 **A set of snapshots of the action potential during the atrial reentrant arrhythmia viewed from the outside of the right atrium.** The normal sinus rhythm beat (*S1*, ‘stimulus’) was initiated in the SAN at 39 ms. The action potential broke out from the SAN to the atrium at the crista terminalis (75 ms). In the opposite direction, the action potential was blocked in the block zone and propagated around the block zone to reach the interatrial septum at 90∼117 ms. The *S2* stimulus at the superior vena cava was delivered at 159 ms. The first reentry wave started on the superior vena cava near the top of the SAN (175.5 ms). The action potental propagated around the SAN (202.5 ms and 211.5 ms) due to the SAN's long refractory period. The second reentry wave started at a similar position and another SAN wave was initiated in the low part of the SAN (247.5 ms). These two waves moved towards each other and finally merged (267∼301.5 ms). Then the third reentry wave started, while the wave in the SAN had not faded away (367 ms). Similar as the first reentry wave, the third wave did not stimulate the SAN due to the long refractory period the SAN.
(TIF)

Figure S3 **A set of snapshots of the action potential during the atrial reentrant arrhythmia viewed from inside of the right atrium.** The action potential propagated faster along the crista terminalis and the main branch than in the rest of the atrial wall (90 ms). The action potential propagated around the block zone to reach the interatrial septum. It then reached the compact node (fast pathway) and inferior nodal extension (slow pathway) (108 ms∼120 ms) at same time. The first reentry wave propagated around the SAN to the interatrial septum due to the SAN's long refractory period (202.5 ms, 207 ms). The wave front reached the compact node (fast pathway) (220 ms) first. It then reached the inferior nodal extension (slow pathway) by passing below the coronary sinus (238.5 ms). The first reentrant wave failed to propagate along the penetrating bundle (277 ms, 300 ms) due to the long refractory period of the penetrating bundle. The second reentrant wave propagated to the interatrial septum earlier than the right atrial free wall (300 ms, 315 ms). The wave front reached the penetrating bundle earlier than the inferior nodal extension and propagated successfully along the penetrating bundle (375 ms).
(TIF)

Movie S1 **Dorsal right view of first reentrant circuit located on the SVC and second reentrant circuit located on the IVC.**
(MP4)

Movie S2 **Dorsal left view of first reentrant circuit located on the SVC and second reentrant circuit located on the IVC.**
(MP4)

Movie S3 **Left oblique view of third reentrant circuit located on the atrial free wall.**
(MP4)

Model S1 **MATLAB workspace file (3D anatomically-detailed model).** Resolution is 60 µm×60 µm×60 µm. The digits in the model signify different tissues: 1 – Outer fatty and connective tissue. 2 – Aortic valve. 3 – Tricuspid valve. 4 – Bicuspid (mitral) valve. 5 – Atrial muscle. 6 – Ventricular muscle. 7 – Coronary sinus. 8 – Fossa ovalis. 9 – Superior vena cava. 10 – Inferior vena cava. 11 – Inferior nodal extension. 12 – Central fibres body. 13 – Sinoatrial node. 14 – Crista terminalis. 15 – Roof bundle. 16 – Main brunch. 17 – Penetrating bundle. 18 – Block zone.
(MAT)

Model S2 **MATLAB workspace file (3D array for simulations).** Resolution is 60 µm×60 µm×60 µm. The digits in the model (Array) signify different tissues: 1 – Atrial wall (isotropic). 2 – Inferior nodal extension (AVN, isotropic). 3 – Sinoatrial node (SAN, anisotropic). 4 – Crista terminalis (anisotropic). 5 – Roof bundle (anisotropic). 6 – Main brunch (anisotropic). 7 – Block zone (passive atrial tissue). 8 – Penetrating bundle (AVN, isotropic). Fibre orientations: (Alpha, Beta, Gamma). (2, 0, 0) in (Alpha, Beta, Gamma) indicate isotropic tissue.
(MAT)
