# Supplementary figures and images for: Three-Dimensional Computer Model of the Right Atrium Including the Sinoatrial and Atrioventricular Nodes Predicts Classical Nodal Behaviours
Source: PLoS One. 2014 Nov 7;9(11):e112547. doi: 10.1371/journal.pone.0112547 (PMC4224508; doi:10.1371/journal.pone.0112547)

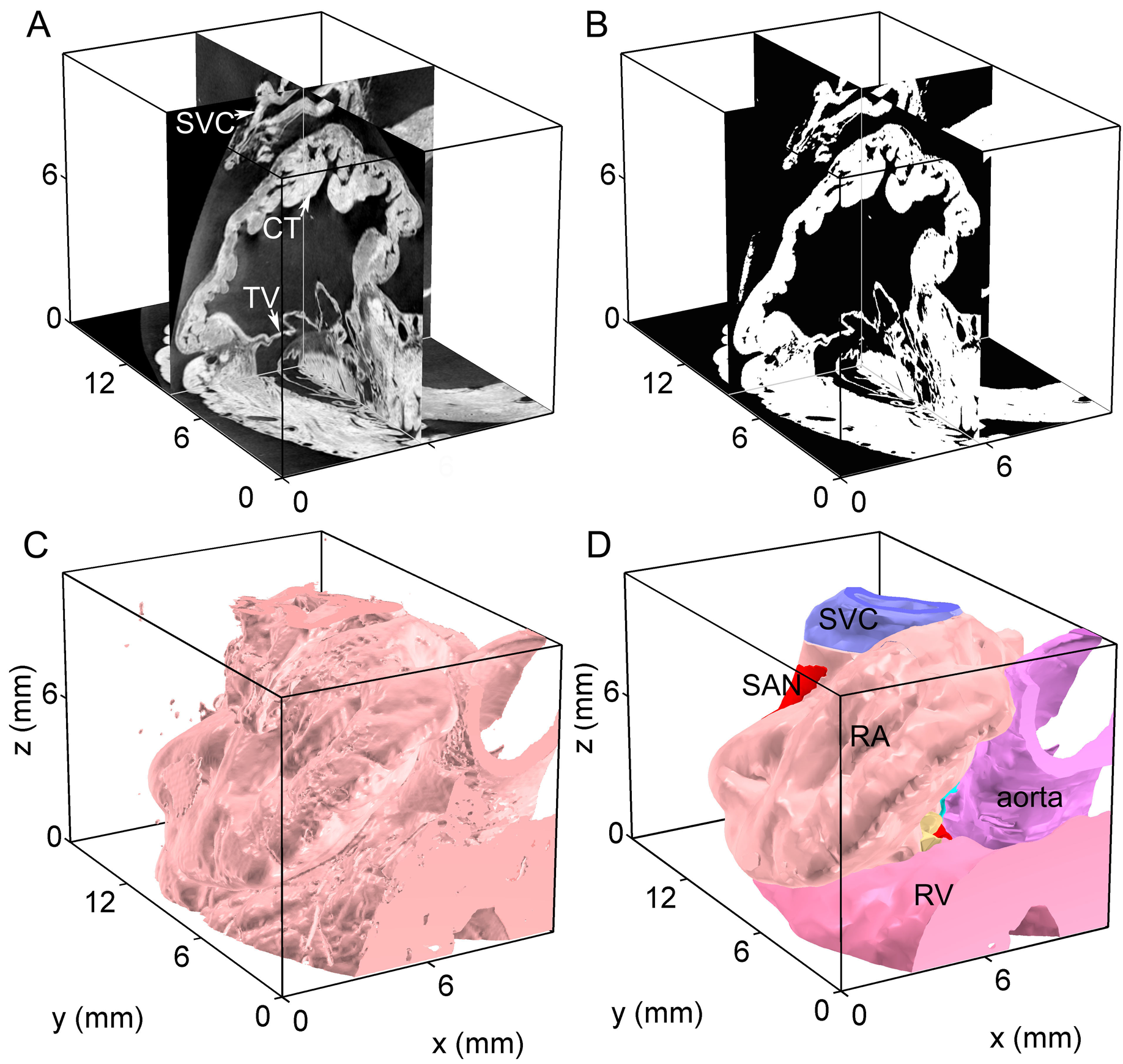

Supplement: Figure S1 — 3D segmentation. A, isotropic 3D MR images; B, converted 3D binary images; C, the 3D model before segmentation; D, the 3D model after segmentation. CT, crista terminalis; RA, right atrium; RV, right ventricle; SAN, sinoatrial node; SVC, superior vena cava; TV, tricuspid valve. (TIF) [file pone.0112547.s001.tif]

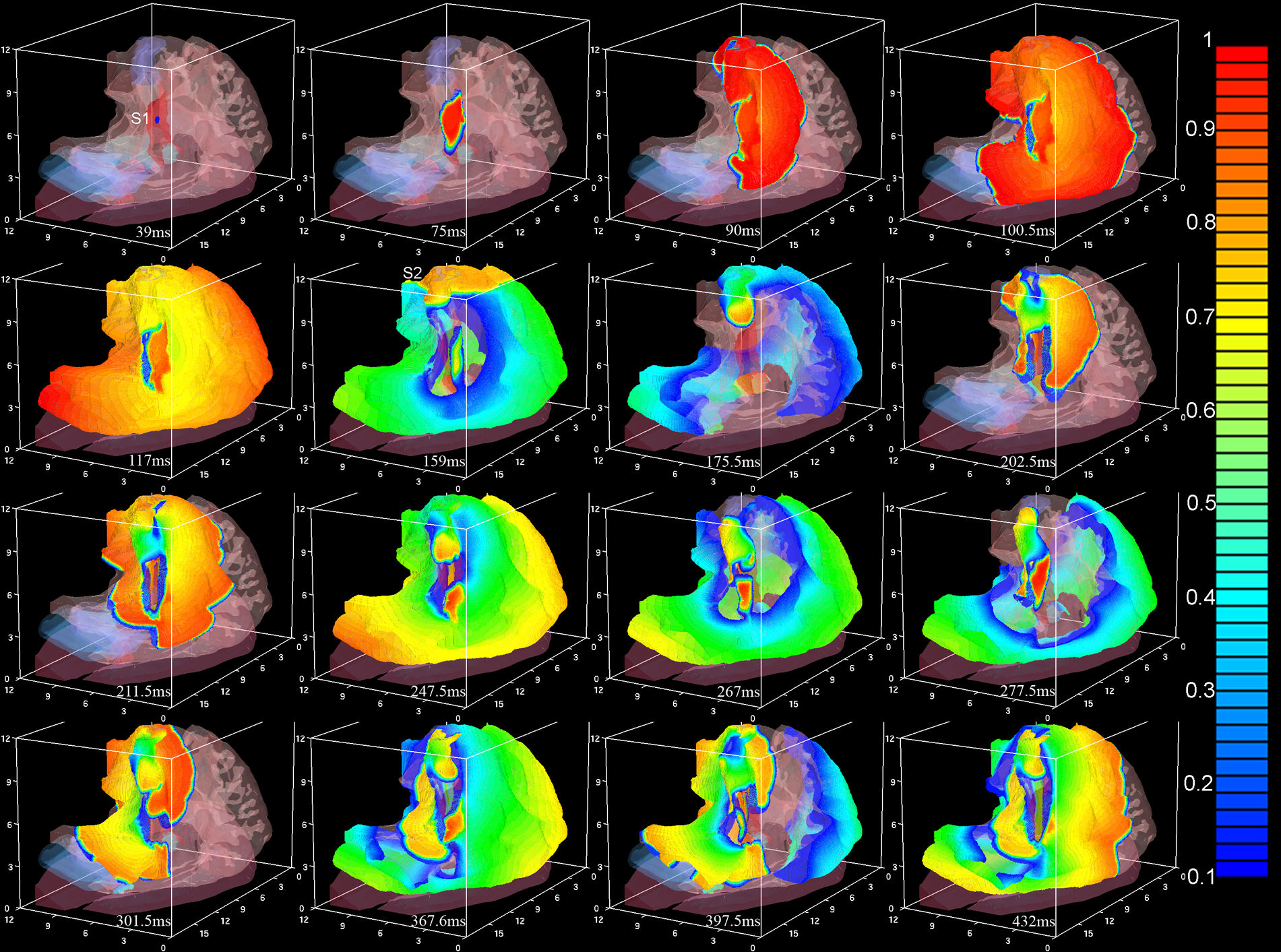

Supplement: Figure S2 — A set of snapshots of the action potential during the atrial reentrant arrhythmia viewed from the outside of the right atrium. The normal sinus rhythm beat (S1, ‘stimulus’) was initiated in the SAN at 39 ms. The action potential broke out from the SAN to the atrium at the crista terminalis (75 ms). In the opposite direction, the action potential was blocked in the block zone and propagated around the block zone to reach the interatrial septum at 90∼117 ms. The S2 stimulus at the superior vena cava was delivered at 159 ms. The first reentry wave started on the superior vena cava near the top of the SAN (175.5 ms). The action potental propagated around the SAN (202.5 ms and 211.5 ms) due to the SAN's long refractory period. The second reentry wave started at a similar position and another SAN wave was initiated in the low part of the SAN (247.5 ms). These two waves moved towards each other and finally merged (267∼301.5 ms). Then the third reentry wave started, while the wave in the SAN had not faded away (367 ms). Similar as the first reentry wave, the third wave did not stimulate the SAN due to the long refractory period the SAN. (TIF) [file pone.0112547.s002.tif]

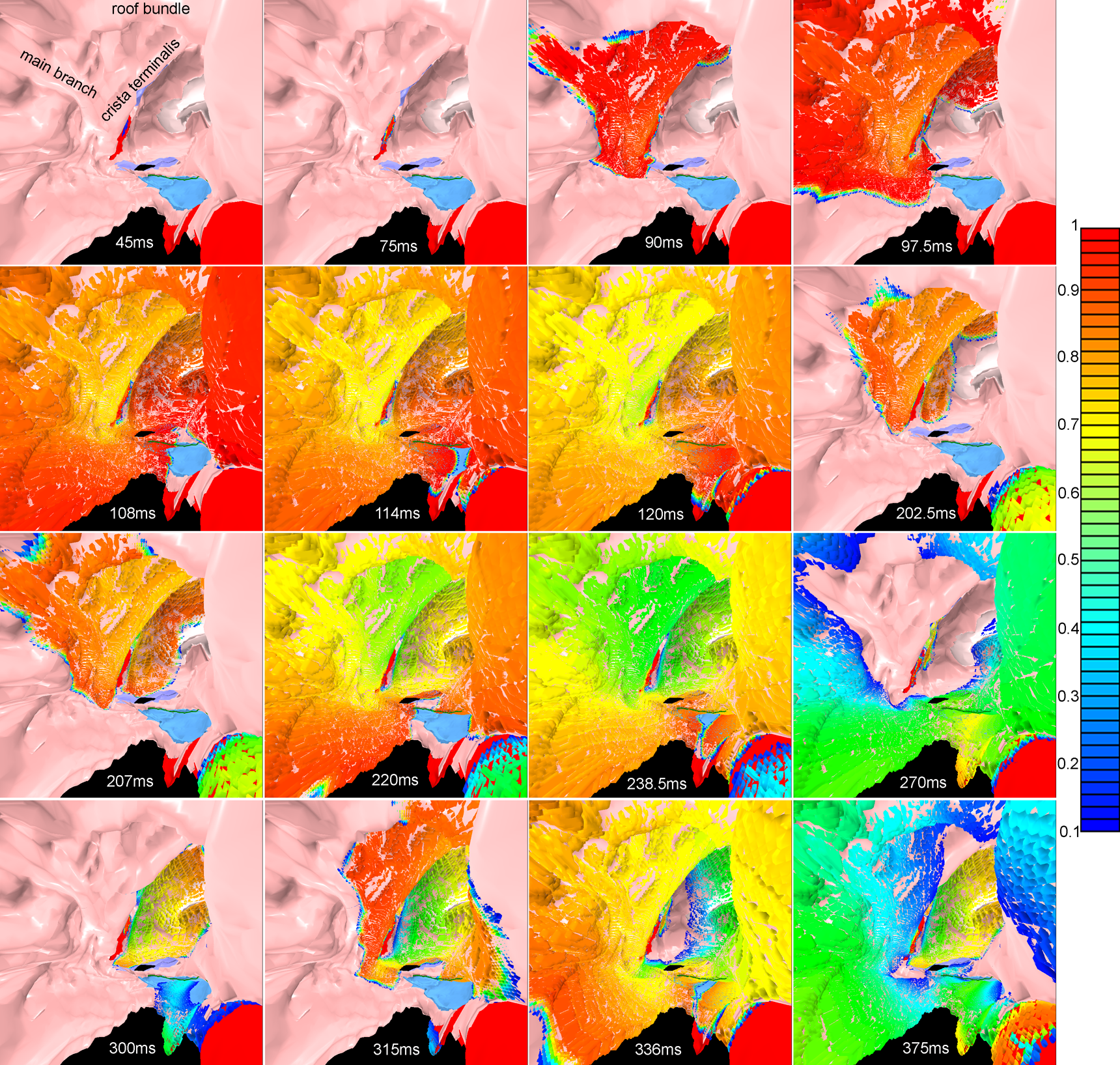

Supplement: Figure S3 — A set of snapshots of the action potential during the atrial reentrant arrhythmia viewed from inside of the right atrium. The action potential propagated faster along the crista terminalis and the main branch than in the rest of the atrial wall (90 ms). The action potential propagated around the block zone to reach the interatrial septum. It then reached the compact node (fast pathway) and inferior nodal extension (slow pathway) (108 ms∼120 ms) at same time. The first reentry wave propagated around the SAN to the interatrial septum due to the SAN's long refractory period (202.5 ms, 207 ms). The wave front reached the compact node (fast pathway) (220 ms) first. It then reached the inferior nodal extension (slow pathway) by passing below the coronary sinus (238.5 ms). The first reentrant wave failed to propagate along the penetrating bundle (277 ms, 300 ms) due to the long refractory period of the penetrating bundle. The second reentrant wave propagated to the interatrial septum earlier than the right atrial free wall (300 ms, 315 ms). The wave front reached the penetrating bundle earlier than the inferior nodal extension and propagated successfully along the penetrating bundle (375 ms). (TIF) [file pone.0112547.s003.tif]
